# Case report: A case of proliferative glomerulonephritis with monoclonal kappa-light chain deposits treated with daratumumab combination therapy

**DOI:** 10.3389/fmed.2024.1462199

**Published:** 2024-10-02

**Authors:** Jue Wang, Jun-Ting Lv, Dan Xiao, Jia Liu, Jun Du, Lu Zhong

**Affiliations:** ^1^Department of Clinical Medicine, Shanghai Jiao Tong University School of Medicine, Shanghai, China; ^2^Zhuhai Hospital of Integrated Traditional Chinese and Western Medicine, Zhuhai, China; ^3^Department of Hematology, Renji Hospital, School of Medicine, Shanghai Jiao Tong University, Shanghai, China

**Keywords:** PGNMID, daratumumab, fixed course, case report, treatment regimen

## Abstract

**Introduction:**

Proliferative glomerulonephritis with monoclonal immunoglobulin deposits (PGNMID) is a chronic glomerular disease caused by monoclonal gammopathy. IgG (mainly IgG3) is the most commonly involved isotype of PGNMID. Here we illustrated a novel medication regimen for the rare variant of PGNMID with deposition of monoclonal immunoglobulin light chain only (PGNMID-LC). Daratumumab has been proved effective in the treatment of plasma cell myeloma while its effect for PGNMID-LC has rarely been reported.

**Methods:**

A daratumumab combination therapy (D-VCd regimen, specifically are daratumumab + dexamethasone + bortezomib + cyclophosphamide) was adopted to treat a patient diagnosed with PGNMID-LC.

**Results:**

The utility of D-VCd regimen showed a favorable effect in this patient. After the fixed course, his clinical symptom, laboratory parameters, neoplastic plasma cells clonity all restored to normal range, and no obvious disease progression was observed throughout the treatment. After a follow up of 14 months, no significant renal or hematological disease progression has been observed.

**Conclusion:**

This case underscores the utility of D-VCd regimen in treatment of PGNMID-LC, and it’s inferred that daratumumab regimen has clinical effects in the disease primarily through targeting tumor clonity. However, data on the use of daratumumab (either in monotherapy or in combination) in clinical trials of PGNMID-LC is currently so limited that that more experiments are needed to support the inference.

## Introduction

Proliferative glomerulonephritis with monoclonal immunoglobulin deposits (PGNMID) is a specific type of monoclonal gammopathy of renal significance (MGRS) where monoclonal immunoglobulins deposit in the glomeruli, leading to proliferative glomerulonephritis. It is characterized by glomerular injury and inflammation due to the deposition of monoclonal IgG, IgA, or rarely IgM. The glomerular deposits are nonorganized with granular amorphous texture and primarily consist of a certain complete Ig molecule with light-chain restriction and heavy-chain subclass restriction (typically IgG3 with *κ*-light chain). Additionally, these deposits are sometimes mixed with complement components (1). However, rare variants with deposits of light chain only, which is termed as PGNMID-light chain (PGNMID-LC), have also recently been described (2).

As a specific form of MGRS, PGNMID is harder to detect due to its lower rate of circulating clones compared to other types of MGRS, which makes it more likely to be overlooked or incorrectly diagnosed during clinical examinations (3).

Daratumumab, an antibody targeting CD38, has proven effective in treating plasma cell myeloma. Considering the substantial expression of CD38 in plasma cell disorders, it could be a potential yet underexploited treatment for specific types of PGNMID that derived from neoplastic plasma cell clones. This report focuses on a patient with PGNMID-LC, characterized mainly by *κ*-light chain in the glomerular deposits, who exhibited clinical recovery after the administration of daratumumab-combination therapy (D-VCd regimen).

## Case description

This case enrolled in February, 2023 at the Renji Hospital affiliated to Shanghai Jiao Tong University, Shanghai, China. The patient was a 43-year-old man who initially presented with unexplained edema of the eyelids and lower limbs, without any apparent cause or hematuria. Biochemical test showed proteinuria was 4446.7 mg/g by urine protein-creatinine ratio. Serum creatinine was 126 μmol/L. The level of serum free *λ* and *κ*-light chain were both above the range while their ratio remained normal. He was subsequently diagnosed with nephrotic syndrome.

**Figure 1:**
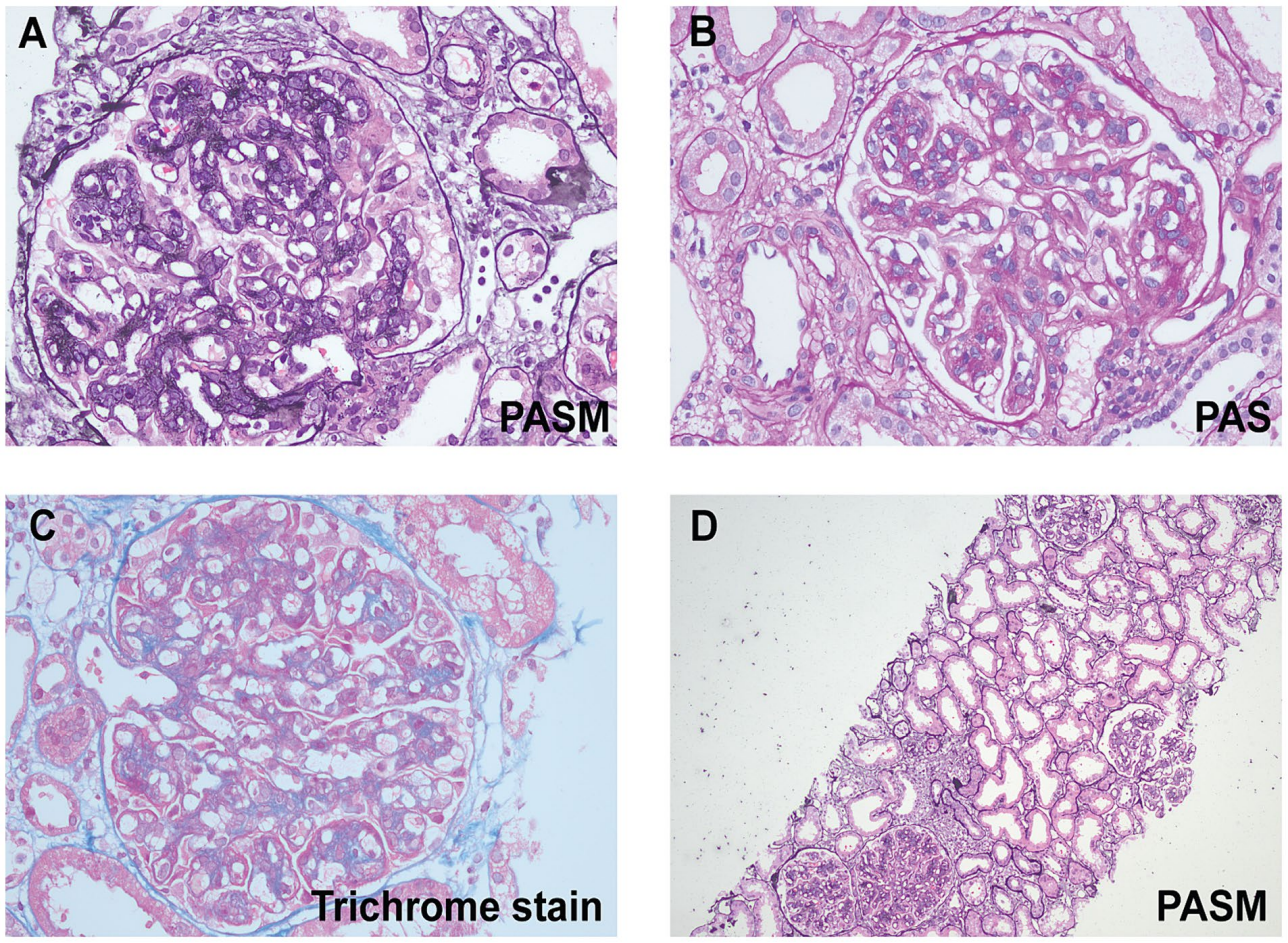
Light microscopic findings. **(A)** High magnification shows lobular appearance of glomeruli due to florid mesangial cell hypercellularity (periodic acid-silver metheramine, original magnification ×400). **(B)** High magnification shows global mesangial hypercellularity and widespread duplication of the glomerular basement membranes. Global, large subendothelial and segmental mesangial nonargyrophilic deposits are seen (periodic acid-Schiff, original magnification ×400). **(C)** A glomerulus on high magnification showing endocapillary hypercellularity and large (wire loop-like) fuchsinophilic subendothelial deposits (trichrome stain, original magnification ×400). **(D)** A low-power image reveals mild vacuolar degeneration in a few renal tubular epithelial cells, with no significant tubular atrophy (periodic acid-silver metheramine, original magnification ×100).

**Figure 2:**
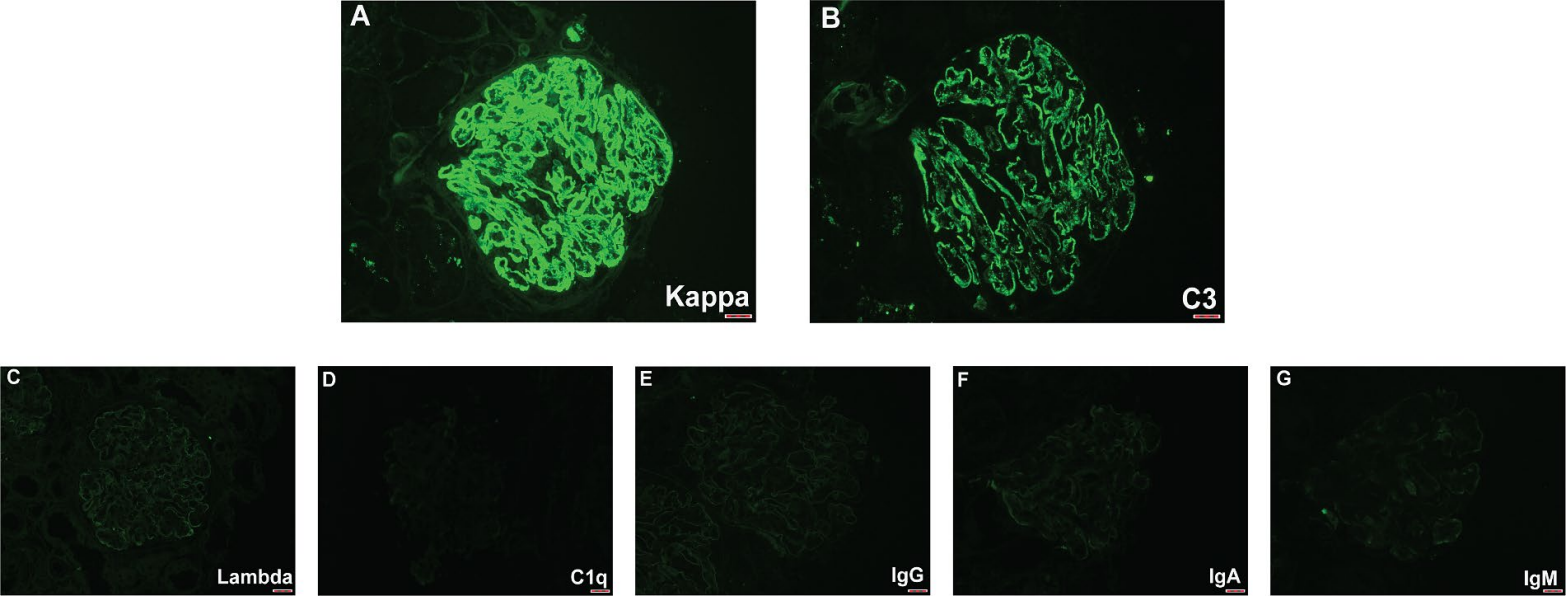
Immunofluorescence findings. **(A)** A high-power figures shows a global granular glomerular capillary wall and mesangial staining for kappa (3+). **(B)** Lower-intensity glomerular staining for C3 was also present (2+). **(C–G)** Glomeruli were negative for lambda, C1q, IgG, IgA, and IgM.

In the renal biopsy, two small pieces of kidney tissue were obtained and some of which were cortical. A total of 9 glomeruli were observed in the entire section, with no significant global or segmental sclerosis detected. In most of the glomeruli, segmental mesangial cell proliferation with an increase in the matrix was evident. Many segments showed thickening of the capillary walls, with some areas showing layering (“tram-track” appearance). In some segments, there was a noticeable increase in the number of circulating cells within the capillaries, leading to poor lumen patency. No obvious loop necrosis or crescent formation was observed. Trichrome staining revealed a substantial amount of fuchsinophilic deposits in the mesangial area and subendothelial space, with fewer deposits in the subepithelial space. A few renal tubular epithelial cells showed mild vacuolar degeneration, with no significant tubular atrophy observed. Some tubules contained protein or red blood cell casts. Interstitial fibrosis or inflammatory cell infiltration was not prominent. No significant necrosis or arteritis was observed in the small arteries (Figure 1). Polarizing microscopy findings were negative. Frozen section staining indicated: IgG1 (−), IgG2 (−), IgG3 (−), IgG4 (−). Paraffin immunofluorescence showed: IgG (−), IgA (−), IgM (−), Kappa (+++), Lambda (−), C1q (−), C3 (++). There was also a small amount of kappa positivity in the cytoplasm of the tubules, while Lambda was negative (Figure 2).

The presumed diagnosis of PGNMID-LC can be established based on light microscope (LM), immunofluorescence (IF) findings, and the clinical presentation, with key features helping distinguish it from other conditions. Amyloid nephropathy (AL) was excluded due to the absence of Congo red-positive amyloid deposits under polarizing microscopy, and the lack of systemic symptoms such as fatigue, weight loss, and multi-organ involvement, which are typically seen in amyloidosis. The patient’s localized nephrotic syndrome, with eyelid and lower limb edema and no systemic manifestations, further made AL unlikely. Fibrillary glomerulonephritis (FGN) can also be ruled out due to its absence of the classical “tram-track” appearance under LM and negative IF for polyclonal IgG. Also, FGN often presents with hematuria and hypertension, our patient had nephrotic syndrome without these features, making FGN less likely. Immunotactoid glomerulopathy (ITG), despite its similarity to PGNMID under LM, was excluded due to the lack of immune complex deposits involving IgG, IgM, or IgA under IF. Besides, ITG is also frequently associated with hematuria and systemic disorders like chronic lymphocytic leukemia (CLL) or lymphoma, which were absent in this patient. Type I cryoglobulinemic glomerulonephritis (CryoGN) was ruled out because no PAS-positive pseudo-thrombi were observed in the capillaries, and the patient did not present with systemic symptoms such as Raynaud’s phenomenon, purpura, or arthralgia, which are typical of cryoglobulinemia. Lastly, light chain deposition disease (LCDD) was excluded based on the absence of linear light chain deposition on IF, and LCDD is associated with systemic light chain deposition and rapid renal deterioration, neither of which were present in this patient. The patient’s involvement was limited to the kidneys, with no systemic organ damage. Moreover, the bone marrow biopsy revealed only 1.5% mature plasma cells, within the normal range, implying no evidence of active myelodysplasia or significant plasmacytosis. Flow cytometry identified just 1.34% monoclonal plasma cells, effectively ruling out multiple myeloma. This combination of findings strongly supported the diagnosis of PGNMID-LC over other potential conditions.

**Figure 3:**
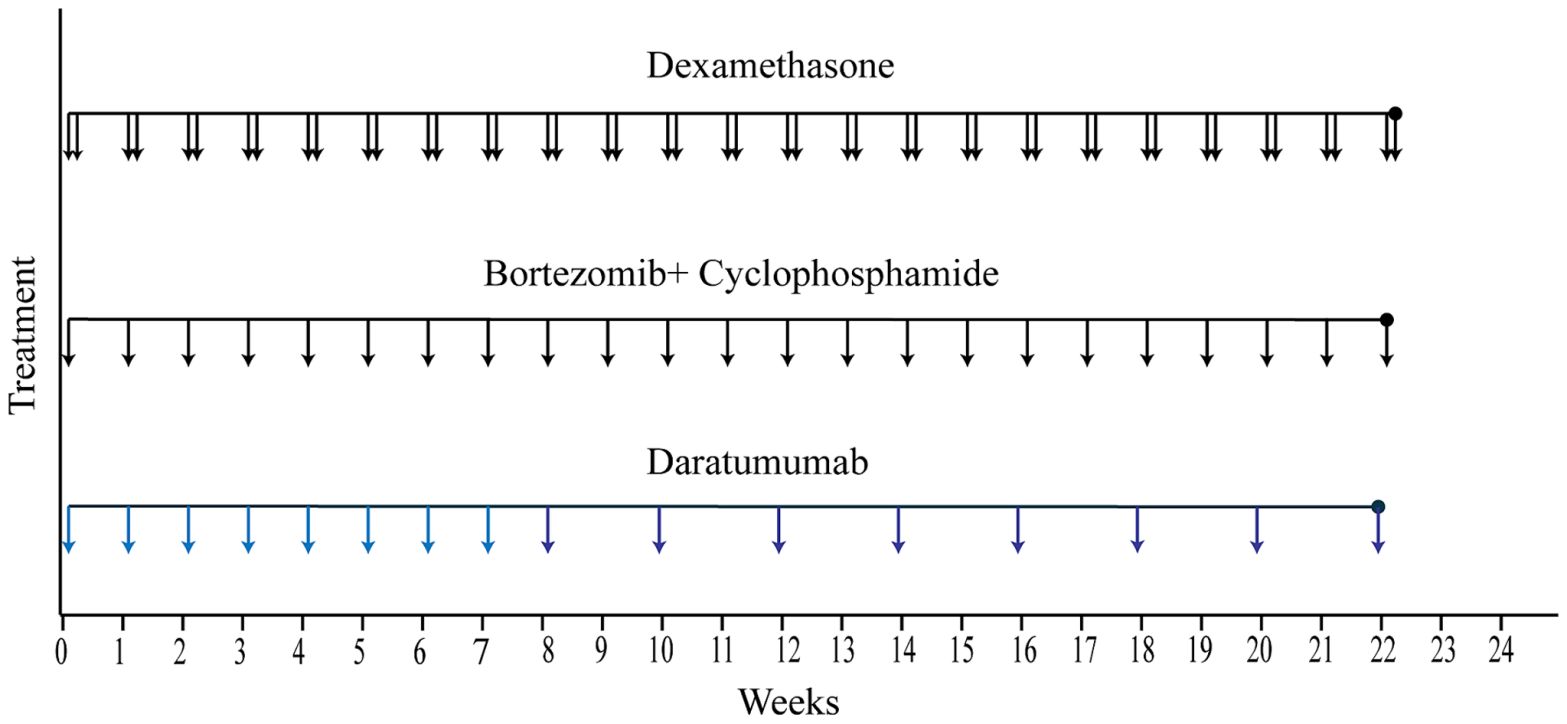
Treatment course. The solid lines represent the treatment course from the time of hospital admission to hospital discharge. (Each vertical arrow represents 20 mg dexamethasone, 2.2 mg bortezomib plus 0.5 g cyclophosphamide, 960 mg daratumumab, respectively).

The initial D-VCd regimen was applied since February, 2023, with a frequency of once a week for the first 8 weeks, and every other week for the next 16 weeks. The specific components of the regimen were daratumumab 16 mg/kg d1 + cyclophosphamide 0.3 g/m^2^ d1 + bortezomib 1.7 mg/ m^2^ d1 + dexamethasone 20 mg d1–d2 qw (the medication of daratumumab was slightly adjusted based on the patient’s weight) (Figure 3).

## Diagnostic assessment

**Figure 4:**
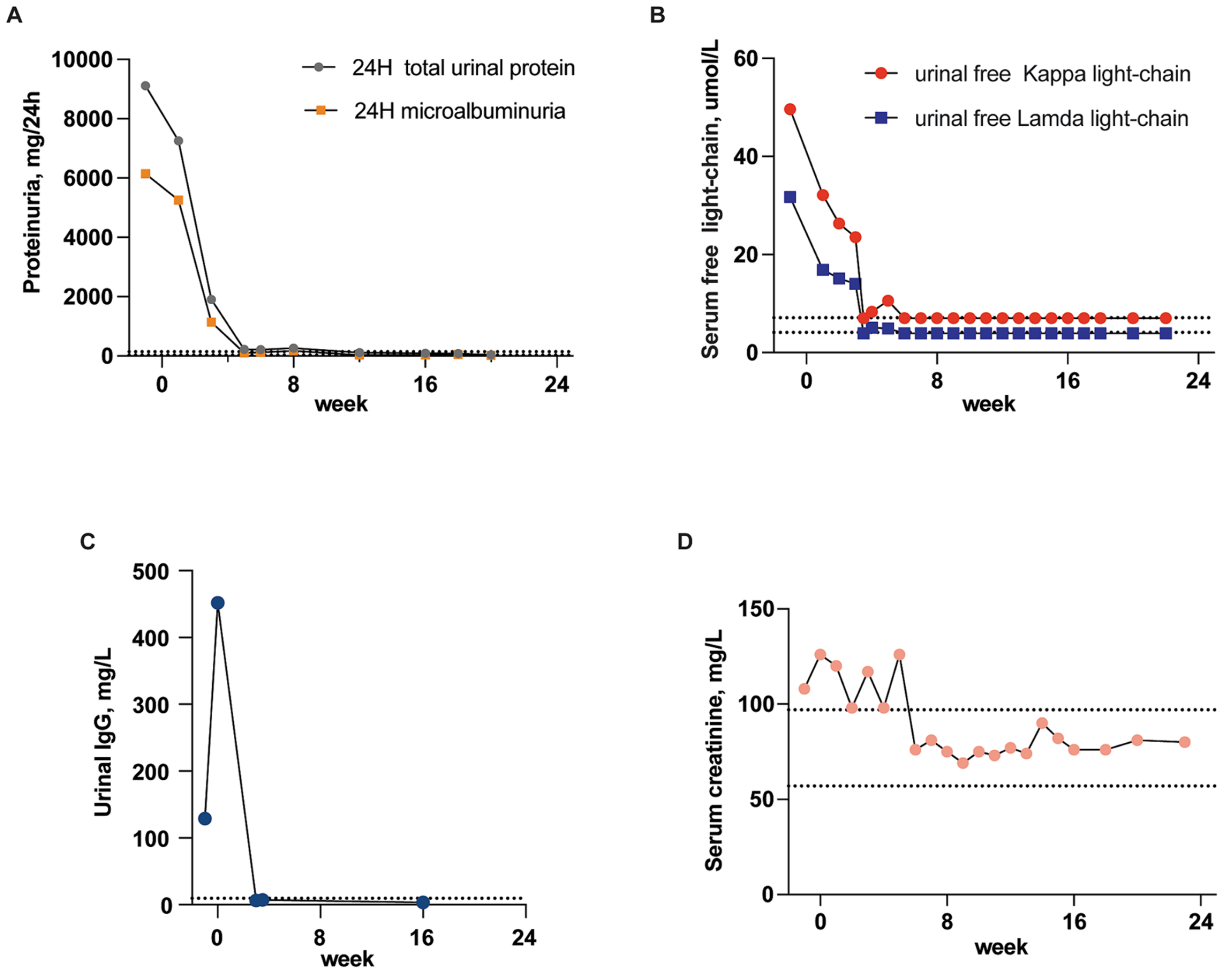
Recovery of renal lesion. Course of the urinal IgG, light chains and protein titer from the time of hospital admission to discharge (the dotted lines indicate normal range). **(A)** Changes in titer of 24H microalbuminuria and total urinary protein. **(B)** Changes in titer of urinal free *κ* and *λ*-light-chain. **(C)** Changes in titer of urinal IgG. **(D)** Serum creatinine fluctuation.

The provided data outlines the patient’s treatment journey and monitors key renal function markers from admission to discharge. The D-VCd regimen proved highly effective for this patient. First of all, the patient experienced symptom relief, notably in the facet of edema reduction. Secondly, lab tests showed a significant reduction in urinal protein, IgG, free *λ* and *κ*-light chain titers. Urinal IgG dropped from 129 mg/L to 6.3 mg/L within 3 weeks, and urinary light chain and protein levels transformed to normal ranges within 6 weeks’ treatment (*κ* and *λ*-light light-chain decreased sharply from 49.6 and 31.7 mg/L to 7 and 3.9 mg/L, respectively, while 24H microalbuminuria and 24H urine total protein levels fell from 6142.35 and 9114.7 mg/24 h to 117 and 212 mg/24 h). These parameters remained within normal range thereafter, implying recovery of renal lesion. Additionally, the patient’s serum creatinine fluctuated within the normal range during the treatment, indicating the renal safety of this regimen (Figure 4). Thirdly, the patient reached great hematologic response. At admission, the bone marrow flow cytometry performed that abnormal plasma cells (marked by CD19–CD38^+^) occupied 1.34% of the total nucleated cells. Of the these neoplastic plasma cells, 93.08% were kappa-restricted, 68.25% were expressed with CD56. Since prior research has identified CD56 as crucial markers for diagnosing, predicting, and monitoring plasma cell disorders (4), the absence of CD56 expression was used as an indicator of the depletion of abnormal plasma cells. Post-chemotherapy bone marrow flow cytometry revealed that the proportion of CD56^+^ cells among nucleated cells was nearly 0%, and the kappa-restriction in plasma cells also disappeared. This indicated the effective removal of abnormal plasma cells and further suggested a normalization of plasma cell clonality (Supplementary Figures S1, S2).

The patient has been followed up for 14 months, no significant renal or hematological disease progression has been observed. The patient’s serum light chains and light chain ratio, urine protein, and creatinine levels are all within the normal range, and the patient has no clinical symptoms of discomfort.

## Discussion

PGNMID-LC is a rare variant of PGNMID, in which the pathogenic deposits consist exclusively of monoclonal immunoglobulin (MIg) LC. Unlike the more common PGNMID-IgG variant, which typically involves IgG, particularly the IgG3 subtype, PGNMID-LC is less understood and less frequently encountered in clinical practice. A multicenter study conducted by Nasr et al. (2) highlighted key differences between PGNMID-LC and PGNMID-IgG that will significantly impact subsequent treatment choices: (i) monoclonal proliferation detection: in PGNMID-LC, 88% of patients have detectable nephropathic clones in the bone marrow, compared to only 9–32% in PGNMID-IgG. (ii) Bone marrow clone type: PGNMID-LC is exclusively associated with plasma cell clones, while PGNMID-IgG may involve plasma cell, lymphocytic, or lymphoplasmacytic clones.

Since the majority of PGNMID-LC patients have detectable nephropathic clones in the bone marrow, targeted therapy against these abnormal hematologic clones is feasible. For this patient, who had a CD38^+^ CD19^−^ abnormal plasma cell clone, a D-VCd regimen was selected, and the therapeutic response was favorable. Based on the efficacy criteria used in previous studies on PGNMID-LC, the patient in this case showed great renal response, and achieved complete remission (CR) and complete response (CR) in terms of renal recovery status and hematologic response after chemotherapy. Renal recovery CR was defined as (5) the remission of proteinuria to <0.5 g/d with normal serum creatinine levels. Hematologic CR was defined according to the International Society of Amyloidosis criteria as (6) negative serum and urine immunofixation with a normal free light chain ratio. Renal response was defined according to the International Society of Amyloidosis criteria as (7) a ≥ 50% reduction (≥0.5 g/d) in 24-h proteinuria with a ≤ 25% decrease in estimated glomerular filtration rate.

While there have been no prior PGNMID cases treated with this exact regimen, many other clonality-directed therapies have been used, some of which included daratumumab. In the study by Nasr et al. (2), 17 patients were included, 12 of whom were diagnosed with *κ* PGNMID-LC. Among them, 3 patients achieved the best response in three key aspects mentioned above. Their treatment regimens were as follows: 1st patient: lenalidomide + dexamethasone, followed by autologous stem cell transplantation (ASCT); 2nd line: bortezomib + bexamethasone + lenalidomide. 2nd patient: cyclophosphamide + bortezomib +dexamethasone + plasmapheresis, followed by ASCT; 2nd line: lenalidomide + dexamethasone + daratumumab. 3rd patient: vincristine + melphalan + dexamethasone, followed by ASCT; 2nd line: lenalidomide + dexamethasone. Despite differences in treatment regimens, these three patients all received ASCT as part of their first-line therapy, combined with plasma cell-directed chemotherapy to inhibit the growth and survival of the pathogenic plasma cells through various mechanisms. In contrast, another 3 patients involved in this study who did not receive plasma cell-directed chemotherapy progressed to end-stage renal disease (ESRD), reinforcing the importance of targeting clonal plasma cells in treating PGNMID-LC. Similarly, for PGNMID-IgG patients with detectable clones, targeted therapy against abnormal hematologic clones is also applicable. A case report (8) described a patient diagnosed with PGNMID with monoclonal deposits of IgG3 with kappa light chain restriction. Flow cytometry of the peripheral blood showed a significant plasma cell population (30% of the B cell compartment) with high CD38 expression but no clear clonal characteristics. The patient was treated with ramipril, amlodipine, and daratumumab, and showed significant improvement after the completion of the treatment course. Another case report (9), involved a patient diagnosed with PGNMID-IgG who did not respond well to steroids combined with valsartan after 1 month. Flow cytometry analysis of the patient’s B-cell subpopulations and immunophenotyping suggested that the IgG deposits might originate from CD38^+^ B cell clones. A following decision was made to administer daratumumab, which led to significant improvement in renal function from the first dose.

To manage PGNMID, the primary goal is to protect and restore renal function and prevent the need for repeat kidney transplantation. Symptomatically, when renal function is compromised or biopsy findings suggest disease progression, PGNMID should be treated with therapy that can alleviate glomerulopathy, using agents such as conventional conservative therapy and non-clone directed drugs, including corticosteroids and mycophenolate mofetil. Sometimes the immunomodulatory drugs (IMiDs) are also administered (3). However, as these therapies are often associated with significant side effects and uncertain effectiveness, some PGNMID patients with less severe renal damage will choose not to take these drugs. In the case report describing a patient diagnosed with PGNMID with monoclonal deposits of IgG3 with kappa light chain restriction, considering the patient’s relatively preserved renal function and mild overall condition (serum creatinine 82 μmoL/L, urine protein to creatinine ratio 816 mg/mmol at admission), the traditional therapies with some undisirable side effects were not utilized (8). Causally, Gumber et al. (10) demonstrated that clone-directed therapy is superior to traditional treatment in PGNMID management. However, the challenge with clone-directed therapy is that circulating paraproteins and pathogenic clones are often undetectable, making treatment selection difficult.

For the majority of PGNMID cases, where nephropathic clones are undetectable (only 30% have identifiable nephropathic clones) (11), treatment strategies generally fall into three categories: conservative therapy, non-clone-directed therapy, and clone-directed therapy (12). Conservative therapy involves the use of renin-angiotensin-aldosterone system inhibitors without specific treatment for MGRS. Researches (5, 12) indicate that spontaneous remission of proteinuria is possible, even in patients with nephrotic-range proteinuria, making conservative therapy a viable option for selected patients with stable renal function and low interstitial fibrosis/tubular atrophy (IFTA). Non-clone-directed therapy, which includes glucocorticoids, oral cyclophosphamide, and mycophenolate mofetil, has been shown to be far less effective than clone-directed therapy (13). Clone-directed therapy includes plasma cell clone (PC)-directed therapy, lymphocytic clone (LC)-directed therapy, and IMiDs. PC-directed therapy can include single agent daratumumab versus bortezomib based regimen. LC-directed therapy includes rituximab. IMiDs include thalidomide, lenalidomide. If conservative therapy fails, targeted therapy against neoplastic clones can be initiated. If no favorable response is observed after 2 or 3 cycles of targeting one clone, therapy should be switched to target other clones (14). According to the report by Zhou et al. (13), there was no statistically significant difference in renal prognosis between patients treated with clone-directed therapies. However, it is important to note that IMiDs have certain limitations compared to bortezomib or daratumumab-based regimens. They are associated with a relatively high incidence of severe side effects and are less well-tolerated in patients with renal insufficiency. Despite these limitations, the high cost of bortezomib or daratumumab may render these options unaffordable for patients with PGNMID in developing countries. In such settings, where financial constraints are a significant factor, IMiDs combined with dexamethasone may serve as a more feasible alternative. Besides, a recent open-label phase 2 trial demonstrated that 6 months of daratumumab in 10 patients with PGNMID (all without detectable clones in bone marrow biopsy) resulted in 4 CR and 6 partial responses (PR) within one year, achieving an overall response rate of 100%. Moreover, the study also proved that daratumumab was well-tolerated in patients with PGNMID, exhibiting a favorable safety profile (15). This suggests that single-agent daratumumab may be a promising treatment option for PGNMID without detectable clones. Additionally, to be noticed, among the patients with PGNMID-IgM without detectable clone, a rituximab-based regimen has been proved effective as most IgM-producing cells are CD20^+^ (16).

Although the D-VCd regimen has not been previously used in PGNMID-LC, it is a potent therapeutic approach in various plasma cell dyscrasias. Recent clinical trials and case reports have demonstrated its efficacy in multiple myeloma (MM) (17), AL (18) and MM-related glomerulopathy (19).

The D-VCd regimen is comprised of two clone-directed drugs (daratumumab and bortezomib) and two non-clone-directed drug (cyclophosphamide and dexamethasone). Daratumumab targets CD38 on clonal plasma cells, inducing their destruction through mechanisms such as antibody-dependent cellular cytotoxicity (ADCC), complement-dependent cytotoxicity (CDC), and direct apoptosis (20–22). This reduces the production and deposition of pathogenic monoclonal immunoglobulins in the kidneys, alleviating glomerular inflammation and improving renal function. Additionally, daratumumab modulates the immune system by reducing immunosuppressive cell populations, thereby enhancing anti-tumor immunity (23, 24). Bortezomib, a proteasome inhibitor, disrupts protein degradation, leading to the accumulation of misfolded proteins and subsequent apoptosis in cancer cells. Cyclophosphamide works as an alkylating agent that induces cytotoxicity through DNA cross-linking, leading to cell death. Dexamethasone acts as an anti-inflammatory agent, modulating the immune response and reducing inflammation. The D-VCd regimen offers great synergic benefits, including enhanced efficacy through complementary mechanisms and a reduction in monoclonal protein levels. By targeting plasma cells, this combination can synergistically deplete pathogenic plasma cells and reduce monoclonal IgG, potentially leading to better therapeutic responses and improved renal outcomes. In this case, initial bone marrow flow cytometry showed 1.34% abnormal plasma cells, which became undetectable after chemotherapy, indicating the regimen’s effectiveness. However, the regimen also poses significant risks. Hematologic toxicity, including thrombocytopenia, neutropenia, and anemia, is a concern, particularly with bortezomib and cyclophosphamide, which can lead to bone marrow suppression and an increased risk of infections, bleeding, and fatigue. As is observed in the LYRA study (17), which evaluated the efficacy and safety of the D-VCd regimen in MM patients, found that fatigue (59%) and neutropenia (13%) were the most frequent treatment-emergent adverse events (TEAEs). Increased risk of infections is another critical concern, as daratumumab depletes CD38-positive immune cells, further compromising immune function. Also, cardiac and renal toxicity, particularly in patients with pre-existing conditions, is also a risk in daratumumab-combined therapy. Besides, cyclophosphamide and bortezomib can cause gastrointestinal toxicity, which can impact the patient’s overall quality of life and adherence to the treatment. The phase 3 ANDROMEDA study (18) on the D-VCd regimen for immunoglobulin light-chain AL showed that D-VCd group led to a higher rate of serious TEAEs compared to VCd group (43.0% vs. 36.2%). The most common grade 3 or 4 adverse events included lymphopenia, pneumonia, cardiac failure, diarrhea, and syncope. Additionally, dexamethasone carries the risk of long-term steroid-related side effects, including hyperglycemia, hypertension, and osteoporosis. Bortezomib is also associated with peripheral neuropathy, which can be dose-limiting and significantly impact the patient’s quality of life. To be noticed, the financial burden of the D-VCd regimen is significant, particularly due to the cost of daratumumab, which may limit access for some patients. In this case, while the D-VCd regimen successfully eradicated abnormal plasma cells, bone marrow flow cytometry revealed that the proportion of normal plasma cells also decreased significantly, from 0.27% to just 0.0327%. Additionally, bone marrow biopsy showed a reduction in the proportion of mature plasma cells, dropping from 1.5 to 0.5%. This highlights the need for close monitoring of patients for potential secondary infections during follow-up.

Therefore, while the D-VCd regimen offers a powerful therapeutic option for neoplastic plasma cell-derived PGNMID, its benefits must be carefully weighed against the potential risks. Daratumumab-combination treatments should be approached carefully. Individualized treatment plans, close monitoring, and supportive care are crucial for optimizing patient outcomes and minimizing harmful side effects. Further clinical studies are needed to better define the role of daratumumab-combination treatment in PGNMID, as well as its long-term outcomes and safety profile.

Finally, it is important to note that one limitation of this report is the lack of electron microscopy (EM) in the diagnostic evaluation due to technical constraints and the restricted biopsy material. EM plays a role in identifying ultrastructural features, such as the characteristic hump-shaped subepithelial deposits typical of PGNMID-LC, which helps better distinguishing it from other glomerulopathies. Although in this case we were able to reach a presumed diagnosis of PGNMID-LC through clinical presentation, LM, and IF, the diagnosis was largely reached by exclusion. Rigorously speaking, the diagnosis of PGNMID-LC should be considered presumptive, as we were unable to confirm the ultrastructural nature of the immune deposits conclusively.

But from another perspective, the technical demands of EM make it inaccessible in some regions, particularly in smaller or resource-limited healthcare settings. This case can provide insights in how physicians can arrive at a presumptive diagnosis of PGNMID-LC without EM, providing useful guidance for treatment in settings where EM is not readily available.

## Data Availability

The raw data supporting the conclusions of this article will be made available by the authors, without undue reservation.
