# Supplementary material for: Case report: A case of proliferative glomerulonephritis with monoclonal kappa-light chain deposits treated with daratumumab combination therapy
Source: Front Med (Lausanne). 2024 Oct 2;11:1462199. doi: 10.3389/fmed.2024.1462199 (PMC11479900; doi:10.3389/fmed.2024.1462199)
Supplement: Supplementary file 1 [file Data_Sheet_1.PDF]

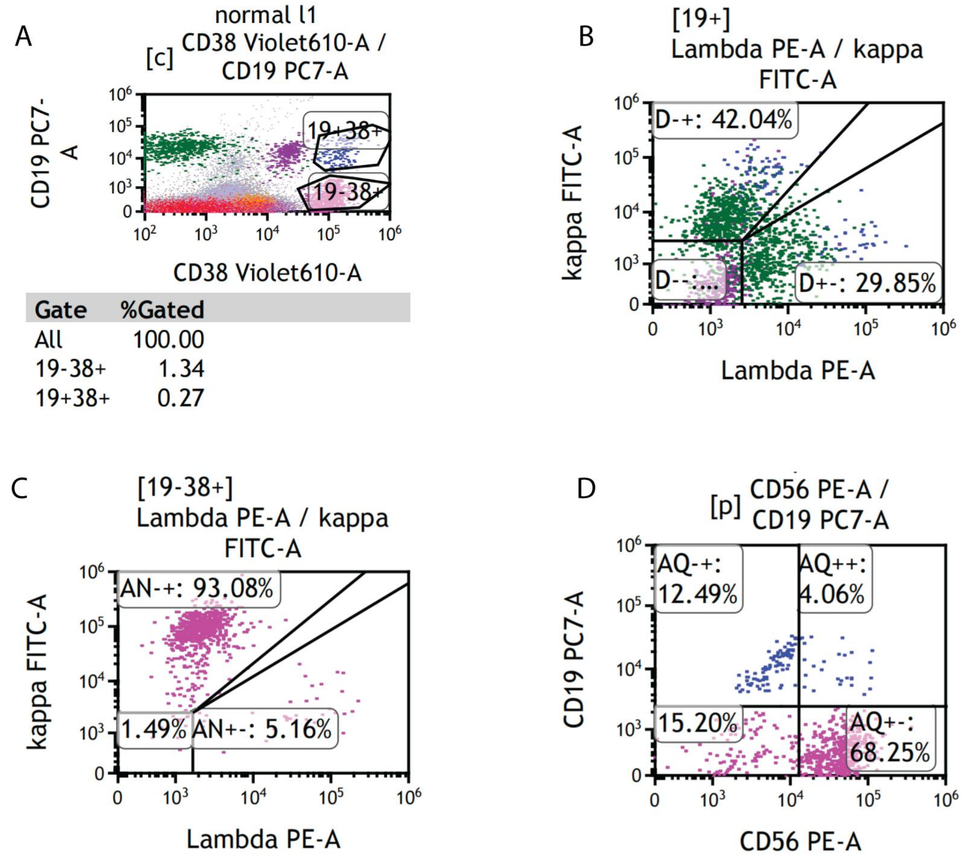

Gate	%Gated
All	100.00
19-38+	1.34
19+38+	0.27

Figure S1. *Findings in flow cytometry analysis before chemotherapy. A. 1.34% of the total nucleated cells were abnormal plasma cells, expressed with CD19-CD38+; B. Of all the CD19+ cells, 42.04% were kappa-restricted; C. of all the CD19-CD38+ cells, 93.08% were kappa-restricted; D. of all the plasma cells*, 68.25% are expressed with CD56.

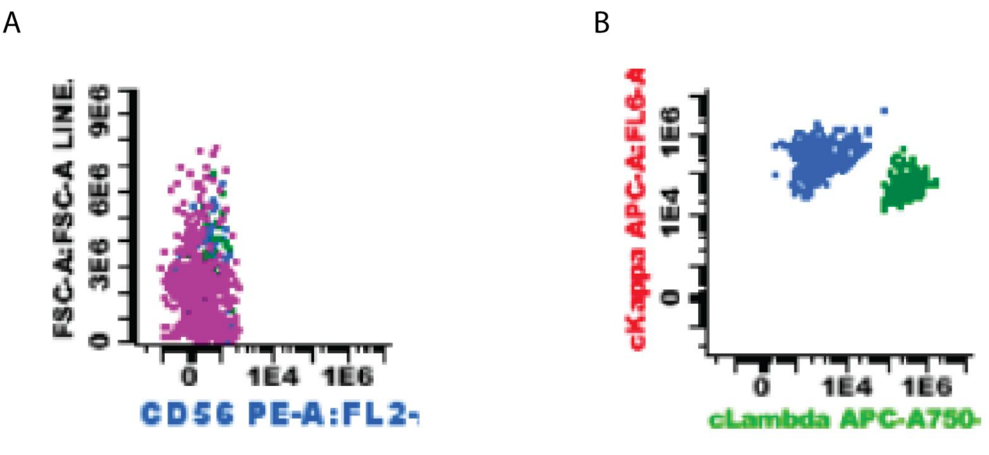

Figure S2. *Findings in flow cytometry analysis after chemotherapy. A. The proportion of CD56+ cells among nucleated cells was nearly 0%, B. kappa-restriction in plasma cells disappeared*
